# Adaptive Neural Backstepping Terminal Sliding Mode Control of a DC-DC Buck Converter

**DOI:** 10.3390/s23177450

**Published:** 2023-08-27

**Authors:** Xiaoyu Gong, Juntao Fei

**Affiliations:** 1 Jiangsu Key Laboratory of Power Transmission and Distribution Equipment Technology, College of Information Science and Engineering, Hohai University, Changzhou 213022, China; 2 College of Artificial Intelligence and Automation, Hohai University, Changzhou 213022, China

**Keywords:** DC-DC buck converter, backstepping control, terminal sliding mode control, double hidden layer recurrent neural network

## Abstract

In this paper, an adaptive backstepping terminal sliding mode control (ABTSMC) method based on a double hidden layer recurrent neural network (DHLRNN) is proposed for a DC-DC buck converter. The DHLRNN is utilized to approximate and compensate for the system uncertainty. On the basis of backstepping control, a terminal sliding mode control (TSMC) is introduced to ensure the finite-time convergence of the tracking error. The effectiveness of the composite control method is verified on a converter prototype in different test conditions. The experimental comparison results demonstrate the proposed control method has better steady-state performance and faster transient response.

## 1. Introduction

With the rapid development of power electronics technology, power electronic converters have been widely used in power systems [1]. Due to its simple structure and high efficiency, the DC-DC buck converter has attracted extensive attention. It has been widely applied in wind energy systems, DC microgrids, DC motor drives, photovoltaic systems and energy storage systems [2,3,4,5,6].

The main control objective of the buck converter is to design a control signal, allowing the output voltage to be adjusted arbitrarily according to the reference value. However, there exist multiple disturbances and uncertainties in the buck converter system, including load variation [7], reference variations [8] and input voltage fluctuations [9], etc. The output voltage regulation has become a challenging task. Hence, how to develop advanced control methods has drawn the attention of researchers.

It can be seen from the early literature that linear control is widely applied in buck converters, such as PI control [10,11] and fixed frequency control [12,13]. Due to simple structure and principle, PI control can obtain accurate tracking without any disturbances. In [12], a fixed-frequency boundary control is designed to maintain the steady state of the buck converter, and Lai et al. [13] developed a fixed-frequency quasi-sliding mode voltage controller. However, both PI control and fixed frequency control possess linear behavior, which makes it difficult to ensure the system’s robustness. Therefore, in recent research, more nonlinear control schemes have been developed for buck converters, such as sliding mode control [14], finite-time control [15] and robust control [16]. As is known, sliding mode control (SMC) is one such nonlinear method that becomes a better choice for converter applications due to its insensitivity to disturbances [17]. In [18], the general design problem of SMC in voltage tracking control is reported, and Tan et al. [19] put an additional double integral term into the sliding surface to suppress the regulation error of the converter.

However, SMC can only ensure that the tracking error asymptotically converges to zero. With regard to this problem, the terminal sliding mode control (TSMC) is proposed, which can obtain finite time convergence of the tracking error by introducing the terminal function. In the buck converter application, Repecho et al. [20] adopted a TSMC algorithm to achieve a faster transient response. Wang and Li [21] designed a discretized fast TSMC algorithm to improve the dynamic properties of the converter system. Babes et al. [22] proposed a new fractional-order nonsingular TSMC method to improve the tracking performance of converters.

The backstepping design has become an effective approach for high-order systems in strict feedback form. It designs virtual control laws for each subsystem via a recursive design process, which can reduce the complexity of the control design [23]. In [24], a single-loop disturbance observer-based control strategy is proposed by using the backstepping technique, and Liu et al. [25] designed a second-order sliding mode control by combining backstepping designs. Here, the backstepping sliding model control (BSMC) is proposed, which is developed in different scenarios. In [26], the BSMC is designed for the position tracking of elastic manipulators with nonlinear elements, and Alam et al. [27] proposed a backstepping integral terminal sliding mode control to obtain voltage control of the buck converter.

Some scholars have investigated model predictive control (MPC). In [28], an MPC is presented for a boost converter to directly regulate output voltage by minimizing the objective function. In [29], a finite control set MPC is proposed to directly control the switching states of the power converter without additional modulation. However, the conventional MPC makes it difficult to reject disturbance and uncertainty, thus causing large estimation errors. Adaptive control is another effective predictive method that can be adopted to estimate model parameters [30]. Xu et al. [31] proposed an adaptive projection algorithm to guarantee the boundness of the estimated term. However, when handling fast-varying parameters, control behavior through adaptive control alone is also not reliable. In addition, several large-signal-based tuning methods are proposed in [32,33,34]. The large signal-averaged model is built by considering the parasitic parameters existing in the model. Specifically, in [32], based on the designed large signal model, an online monitoring technology is utilized to estimate the capacitor and its equivalent series resistance. However, this method fails to deal with the effect of other parameter variations. Leung and Chung [33] proposed a dynamic hysteresis control to guarantee the large-signal stability of a buck converter by setting the state of the main switch. Liu and Sen [34] designed a general large-signal averaged model for a buck converter to eliminate the effect of the parasitic parameters. However, when facing parameter variations, these methods make it difficult to obtain satisfactory results. These shortcomings can be overcome by adopting the neural network feedforward control method.

Since the hidden layer of the neural network adopts the Gaussian function as an activation function, it possesses nonlinear mapping capability and can be utilized to estimate complex nonlinear functions, which provides an effective solution to handle system uncertainty [35]. As a simple and common network structure, the radial basis function neural network (RBFNN) is reported in [36] for handling the uncertain term of the system, but the approximation accuracy of RBFNN is low, and it is difficult to deal with the time-varying inputs. Tousif and Chitralekha [37,38] proposed an adaptive backstepping controller based on a single-layer Chebyshev neural network (CNN). The Chebyshev polynomials are used to approximate the unknown load resistance. In [39,40], adaptive neural networks are designed for nonlinear control, where the neural network parameters are updated online through adaptive laws, and thus the neural network can obtain self-regulation capability. In [41], a neural network controller is designed to rapidly track reference trajectory, but the proposed neural structure is relatively complex, and the requirements for training data are high, so it is not a favorable choice. Meanwhile, the recurrent neural networks (RNN) proposed in [42,43] not only have a simple structure but also possess a higher approximation accuracy due to the addition of a neuron feedback loop. In [42], a self-organizing recurrent radial basis function neural network is developed to predict the future dynamic behaviors of a nonlinear system. In [43], a novel adaptive fuzzy RNN is proposed to solve the nonrepetitive motion problem of robot manipulators. On the other hand, the single-layer neural network may need to train numerous neurons, which will increase the computational complexity. Hence, the multiple layer neural network (MLNN) has been developed in [44,45], and both the reduced neuron training and stronger estimation capability of the neural network can be obtained. Fei et al. [44] designed a multilayer fuzzy neural network for the tracking problem of a class of universal SISO nonlinear systems. In [45], for the harmonic compensation of the active power filter, a multiple hidden layer neural network is proposed to estimate the equivalent control term. There also exist some fuzzy neural controllers that make full use of neural networks to improve the control effect of complex models, reflecting the versatility and structural flexibility of neural networks [46,47,48,49].

More sliding mode control methods based on neural networks are investigated for different application scenarios. In [50], a second-order sliding mode control (SOSMC) using a wavelet fuzzy neural network (WFNN) is proposed for PMSM, where the WFNN is adopted to approximate the lumped uncertainty. Fei et al. [51] developed the fractional sliding-mode control and recurrent neural network (RNN) for the tracking control of the micro-gyroscope system, where the RNN is utilized to estimate the system uncertainty. Moreover, for active power filter (APF), Chen et al. [52] designed an RNN to estimate the switching term gain of sliding mode control, which can effectively avoid the switching gain being selected as too large or too small, and thus the chattering phenomenon can be alleviated.

More recently, some advanced controllers have been investigated to obtain better performance of the buck converter. In [53], a fractional-order SMC with a high-order disturbance observer (DO) is proposed, where two observers are built to estimate both matched and mismatched disturbances to improve the stability and dynamic performance of the converter system. Yang et al. [54] investigated an optimized active disturbance rejection control (ADRC) method, and a new reduced-order generalized proportional integral observer is designed to estimate the lumped disturbances. This control scheme presents strong robustness to various disturbances and uncertainties. Furthermore, according to the passivity theory and coordinate transformation, He et al. [55] developed a new energy-based controller for the buck converter.

Motivated by this research, an adaptive backstepping terminal sliding mode controller using a DHLRNN structure is designed for a buck converter. The main contributions of the proposed strategy are listed as

(1) The DHLRNN can be used to estimate complex nonlinear functions due to its strong learning ability and compensation accuracy. To handle the model uncertainty and improve the tracking performance of the buck converter, the DHLRNN is designed to estimate the nonlinear function of the converter system, and the nonlinear function being estimated integrates state variables and model parameters. The DHLRNN possesses strong learning capability to approximate the nonlinear function;
(2) The ABTSMC is introduced to ensure finite-time convergence and reduce the complexity of the control design. The switching control term can counteract the external disturbances and network approximation error, thus improving the steady-state accuracy and disturbance rejection performance.

## 2. System Description and Problem Statement

### 2.1. DC-DC Buck Converter Model

**Figure 1:**
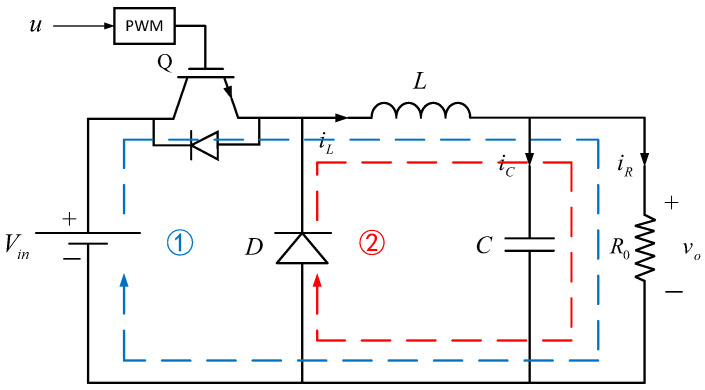
The circuit diagram of the DC-DC buck converter (ON case: Line 1, OFF case: Line 2).

Figure 1 gives the typical circuit diagram of a DC-DC buck converter, where $R_{0}$ denotes a load resistance, L denotes a filter inductor, C denotes a filter capacitor, D denotes a diode, Q denotes a controlled switch (insulated gate bipolar transistor, IGBT) and $V_{in}$ denotes a DC-supplied voltage. The switch ON and OFF cases of the DC-DC buck converter are plotted by dashed lines 1 and 2.

According to the Kirchhoff law and state averaging method, the dynamic model of the DC-DC buck converter can be expressed as

(1) $$\left\{ \begin{array}{l} \frac{di_{L}}{dt}=-\frac{1}{L}v_{o}+\frac{V_{in}}{L}u\\ \frac{dv_{o}}{dt}=\frac{1}{C}i_{L}-\frac{1}{R_{0}C}v_{o} \end{array}\right.$$

where $v_{o}$ and $i_{L}$ represent output voltage and inductor current, respectively. u∈[0,1] is the control signal, i.e., duty ratio, which is compared with a fixed-frequency triangle signal to generate the PWM driving signal.

Considering the output voltage and its derivative as the state variables, it can be obtained that

(2) $$\left\{ \begin{array}{l} x_{1}=v_{o}\\ x_{2}={\dot{x}}_{1}=\frac{dv_{o}}{dt} \end{array}\right.$$

Hence, the dynamic model (1) can be rewritten as

(3) $$\left\{ \begin{array}{l} {\dot{x}}_{1}=x_{2}\\ {\dot{x}}_{2}=f(x)+Fu \end{array}\right.$$

where $f(x)=-\frac{x_{1}}{LC}-\frac{x_{2}}{R_{0}C}$ is the nonlinear function of the system and $F=\frac{V_{in}}{LC}$ is the control coefficient.

**Remark** **1.** *The control coefficient* F *is considered bounded such that* $F_{i}\leq F\leq F_{a}$, *where* $F_{i}$ *and* $F_{a}$ *are positive constants*.

**Remark** **2.** *In a practical system, due to environmental fluctuations and measurement errors, the model parameters are difficult to obtain accurately. Moreover, considering the parasitic components in the converter system, such as inductor/capacitor equivalent series resistance (ESR), these factors will lead to system uncertainty, and thus, the voltage regulation accuracy of the buck converter will inevitably be degraded. Therefore, based on the above uncertainties*, f(x) *and* F *are rewritten as*

(4) $$f(x)=f_{0}(x)+\Delta f(x)$$

(5) $$F=F_{0}+\Delta F$$

*where* $f_{0}(x)$ *and* $F_{0}$ *are the nominal parts*, Δf(x) *and* ΔF *are the uncertain terms, which comprise parameter and parasitic component variations. These terms possess strong nonlinear behavior, and their variations are relatively complex. To handle system uncertainty, the proposed DHLRNN is designed to estimate the nonlinear function* f(x), *and the adaptive law using a projection algorithm is adopted to estimate the control coefficient* F. *Then, the above two estimation methods are integrated into the structure of the proposed control algorithm. Due to the learning and approximation capability of the DHLRNN and adaptive projection algorithm, they can be utilized in real time to counteract and compensate for parameter and parasitic component variations by introducing estimates into the controller*.

### 2.2. Backstepping SMC Design

The main principle of backstepping control is to decompose the complex nonlinear system into subsystems. Then, the virtual control term is recursively designed for each subsystem, and thus, the complexity of the control design can be reduced.

The reference voltage value is denoted as $v_{ref}$. According to the backstepping control theorem, the backstepping variables $z_{1}$ and $z_{2}$ are defined as

(6) $$z_{1}=x_{1}-v_{ref}$$

(7) $$z_{2}=x_{2}+cz_{1}-{\dot{v}}_{ref}$$

where c>0, $z_{2}$ is the virtual control term.

Considering the following Lyapunov function as

(8) $$V_{1}=\frac{1}{2}z_{1}^{2}$$

According to (6) and (7), it can be obtained that

(9) $${\dot{z}}_{1}=z_{2}-cz_{1}$$

Taking the derivative of $V_{1}$ and combing with the dynamic model (3) yields

(10) $${\dot{V}}_{1}=-cz_{1}^{2}+z_{1}z_{2}$$

If $z_{2}=0$, then ${\dot{V}}_{1}\leq 0$ can be obtained. Therefore, $z_{2}\to 0$ will be proved in the following steps.

The sliding surface is defined as

(11) $$s=kz_{1}+z_{2}$$

where k>0 is the sliding gain.

Taking the derivative of s yields:

(12) $$\dot{s}=k(z_{2}-cz_{1})+f(x)+Fu+c{\dot{z}}_{1}-{\ddot{v}}_{ref}$$

Then, the ideal control law u is designed as follows:

(13) $$u=\frac{1}{F}(-k(z_{2}-cz_{1})-f(x)-c{\dot{z}}_{1}+{\ddot{v}}_{ref}-h(s+\beta \mathrm{sgn}(s)))$$

where h and β are positive constants.

**Theorem** **1.** *For the dynamic model of the buck converter (3), if the model parameters are all known, the sliding surface is chosen as (11), and the control law is designed as (13), then the stability of the closed-loop system is guaranteed*.

**Proof** **of** **Theorem** **1.** A new Lyapunov function candidate is defined as

(14) $$V=V_{1}+\frac{1}{2}s^{2}$$

Differentiating V and combining with (13) to obtain

(15) $$\begin{array}{ll} {\dot{V}}_{2} & =-cz_{1}^{2}+z_{1}z_{2}+s(k(z_{2}-cz_{1})+f(x)+Fu+c{\dot{z}}_{1}-{\ddot{v}}_{ref})\\ & =-cz_{1}^{2}+z_{1}z_{2}-hs^{2}-h\beta \left|s\right|\\ & =-z^{T}Qz-h\beta \left|s\right| \end{array}$$

where $z={\left[\begin{array}{cc} z_{1} & z_{2} \end{array}\right]}^{T}$ and

(16) $$Q=\left[\begin{array}{cc} c+hk^{2} & hk-\frac{1}{2}\\ hk-\frac{1}{2} & h \end{array}\right]$$

If Q is a positive definite matrix, it can be concluded that

(17) $${\dot{V}}_{2}=-z^{T}Qz-h\beta \left|s\right|\leq 0$$

As can be seen from (16), $\left|Q\right|=h(c+k)-\frac{1}{4}$, then Q can be guaranteed to be a positive definite matrix through choosing h, c and k.

According to the Lyapunov stability theorem and LaSalle invariance principle, the stability of the closed-loop system can be guaranteed, and the voltage tracking error will asymptotically converge to zero. □

## 3. Design of Adaptive Backstepping Terminal Sliding Mode Control Using DHLRNN

Due to the effect of environmental fluctuations, the exact model parameters are difficult to obtain directly in practical application because it will cause system uncertainty. Therefore, the control law (13) is not reliable. Moreover, the above controller can only guarantee that the voltage tracking error asymptotically converges to zero. To effectively compensate for the system uncertainty and obtain the finite-time convergence of the tracking error, both the TSMC and DHLRNN are integrated into the controller design.

### 3.1. Structure of DHLRNN

**Figure 2:**
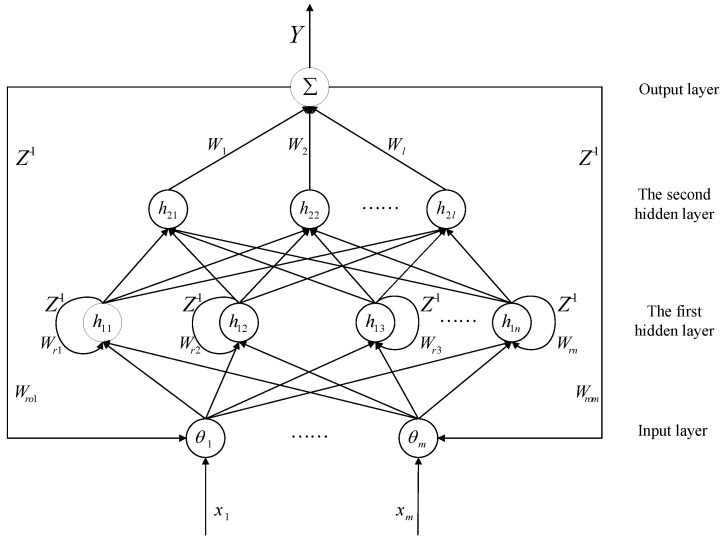
The structure of the DHLRNN.

The four-layer structure of the DHLRNN is shown in Figure 2, where the activation function of each hidden layer adopts the Gaussian function. Moreover, the previous output vector will be fed back to the current input nodes through feedback weights, which can improve the approximation performance and dynamic regulation capability of the neural network. The node input and node output in each layer of the proposed neural network are described as follows:

(1) **Input layer:** In this layer, each node will transmit input data to the subsequent layers, and the previous output value exY from the output layer will be fed back to the current input layer. The ith node output can be described as
  (18) $$\theta_{i}=x_{i}\cdot W_{roi}\cdot exY,(i=1,2,\cdots ,m)$$
  where $X={\left[x_{1},x_{2},\cdots x_{m}\right]}^{T}$ is the input vector, $\theta ={\left[\theta_{1},\theta_{2},\cdots ,\theta_{m}\right]}^{T}$ is the output vector and $W_{ro}={\left[W_{ro1},W_{ro2},\cdots W_{rom}\right]}^{T}$ is the feedback weight vector.
(2) **First hidden layer:** The output form of this layer adopts a nonlinear activation function $\phi_{1j}$, which can map the input signal to a high-dimensional space and extract signal features. The neuron feedback loop is constructed in this layer, and the previous output vector $L\phi_{1j}$ is connected to the current input nodes. Thus, the self-regulation capability of the neural network can be improved through the cyclic connections of the neurons. The jth node output is described as follows:
  (19) $$\begin{array}{c} \phi_{1j}=e^{-net_{1j}},(j=1,2,\cdots ,n)\\ net_{1j}=\sum_{i=1}^{m}\frac{{\left(\theta_{i}\cdot W_{rj}\cdot L\phi_{1j}-c_{1j}\right)}^{2}}{b_{1j}^{2}} \end{array}$$
  where the center vector is $C_{1}={\left[c_{11},c_{12},\cdots c_{1n}\right]}^{T}$, the base width vector is $B_{1}={\left[b_{11},b_{12},\cdots ,b_{1n}\right]}^{T}$, the output vector of this layer is $\Phi_{1}={\left[\phi_{11},\phi_{12},\cdots \phi_{1n}\right]}^{T}$, and the internal feedback weight vector is $W_{r}={\left[W_{r1},W_{r2},\cdots W_{rn}\right]}^{T}$.
(3) **Second hidden layer:** In the second hidden layer, the Gaussian function is also utilized here as the activation function to further implement dynamic mapping and extract signal features. The kth node output is described as follows:
  (20) $$\begin{array}{c} \phi_{2k}=e^{-net_{2k}},(k=1,2,\cdots ,l)\\ net_{2k}=\sum_{j=1}^{n}\frac{{\left(\phi_{1j}-c_{2k}\right)}^{2}}{b_{2k}^{2}} \end{array}$$
  where the center vector is $C_{2}={\left[c_{21},c_{22},\cdots c_{2l}\right]}^{T}$, the base width vector is $B_{2}={\left[b_{21},b_{22},\cdots ,b_{2l}\right]}^{T}$ and the output vector of this layer is $\Phi_{2}={\left[\phi_{21},\phi_{22},\cdots \phi_{2l}\right]}^{T}$.
(4) **Output layer:** This layer has only one node, and each node output of the second hidden layer is connected to the output layer through the weights $W_{k}(k=1,2,\cdots ,l)$. The overall output of the neural network is calculated as
  (21) $$Y=W^{T}\Phi_{2}=W_{1}\phi_{21}+W_{2}\phi_{22}+\cdots +W_{l}\phi_{2l}$$
  where the output weight vector is $W={\left[W_{1},W_{2},\cdots ,W_{l}\right]}^{T}$.

### 3.2. Controller Design and Stability Analysis

**Figure 3:**
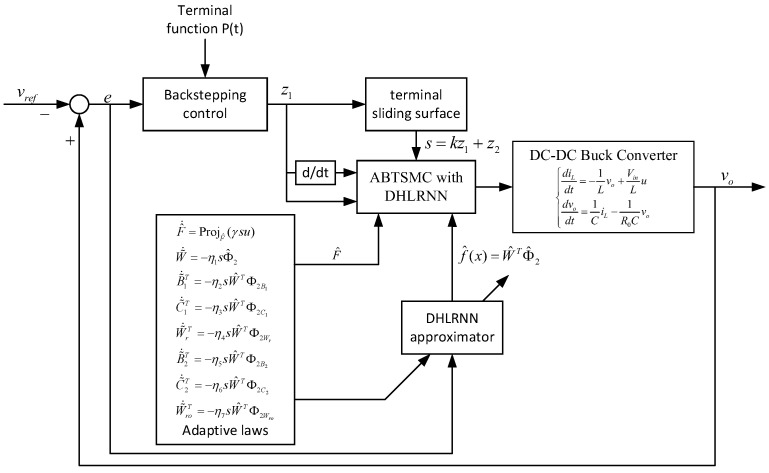
The block diagram of the proposed control method.

The block diagram of the proposed control method is plotted in Figure 3, where the DHLRNN is utilized to estimate the nonlinear function f(x), and the adaptive projection algorithm is adopted to approximate the control coefficient F. Moreover, all parameters of the DHLRNN are trained online through the adaptive laws derived from the Lyapunov stability theorem. Hence, the system uncertainty can be effectively counteracted and compensated by introducing the above estimates into the controller.

To guarantee the finite-time convergence of tracking errors, the terminal function p(t) is introduced. Thus, the backstepping variables $z_{1}$ and $z_{2}$ are redefined as

(22) $$\begin{array}{l} z_{1}=x_{1}-v_{ref}-p=e-p\\ z_{2}=x_{2}+cz_{1}-{\dot{v}}_{ref}-\dot{p} \end{array}$$

**Remark** **3.** *With regard to the definition of the terminal function* p(t), *for the sake of ensuring global robustness of the system, let* e(0)=p(0), $\dot{e}(0)=\dot{p}(0)$. *Moreover, in order to ensure that the tracking error can obtain finite-time convergence, when* t≥T, *the condition of* p(t)=0, $\dot{p}(t)=0$, $\ddot{p}(t)=0$ *should hold. Thus, the terminal function* p(t) *can be constructed as follows*:

(23) $$p(t)=\left\{ \begin{array}{l} e(0)+\dot{e}(0)t+\frac{1}{2}\ddot{e}(0)t^{2}-(\frac{a_{00}}{T^{3}}e(0)+\frac{a_{01}}{T^{2}}\dot{e}(0)+\frac{a_{02}}{T}\ddot{e}(0))t^{3}\\ +(\frac{a_{10}}{T^{4}}e(0)+\frac{a_{11}}{T^{3}}\dot{e}(0)+\frac{a_{12}}{T^{2}}\ddot{e}(0))t^{4}\\ -(\frac{a_{20}}{T^{5}}e(0)+\frac{a_{21}}{T^{4}}\dot{e}(0)+\frac{a_{22}}{T^{3}}\ddot{e}(0))t^{5},0\leq t\leq T\\ 0,t>T \end{array}\right.$$

 *where* $a_{ij}(i,j=0,1,2)$ *are the equation coefficients*.

Then, the sliding surface is defined as follows:

(24) $$s=kz_{1}+z_{2}$$

where $z_{1}$ and $z_{2}$ are the redefined variables from (22), k>0 is the sliding gain.

The derivative of s is given by

(25) $$\begin{array}{ll} \dot{s} & =k{\dot{z}}_{1}+{\dot{z}}_{2}\\ & =k(z_{2}-cz_{1})+f(x)+Fu+c{\dot{z}}_{1}-{\ddot{v}}_{ref}-\ddot{p} \end{array}$$

According to the optimal approximation theory, there exist optimal parameters $W^{*},$ $B_{1}^{*},C_{1}^{*},W_{r}^{*},B_{2}^{*},C_{2}^{*},W_{ro}{}^{*}$ to estimate the nonlinear function such that

(26) $$f(x)=W^{{*}^{T}}\Phi_{2}^{*}(B_{1}^{*},C_{1}^{*},W_{r}^{*},B_{2}^{*},C_{2}^{*},W_{ro}^{*})+\varepsilon$$

where ε is the network reconstructed error.

Then, when the nonlinear function is approximated by the proposed neural network, the corresponding estimated value is expressed as

(27) $$\hat{f}(x)={\hat{W}}^{T}{\hat{\Phi }}_{2}({\hat{B}}_{1},{\hat{C}}_{1},{\hat{W}}_{r},{\hat{B}}_{2},{\hat{C}}_{2},{\hat{W}}_{ro})$$

Hence, the following approximation error of the neural network can be obtained:

(28) $$f(x)-\hat{f}(x)={\tilde{W}}^{T}{\hat{\Phi }}_{2}+{\hat{W}}^{T}{\tilde{\Phi }}_{2}+\varepsilon_{0}$$

where $\varepsilon_{0}={\tilde{W}}^{T}{\tilde{\Phi }}_{2}+\varepsilon$.

Here, Taylor expansion is employed for linearization, and thus, the nonlinear DHLRNN can be transformed into a partial linear form. The expansion of ${\tilde{\Phi }}_{2}$ is expressed as

(29) $$\begin{array}{ll} {\tilde{\Phi }}_{2} & =\Phi_{2B_{1}}\cdot {\tilde{B}}_{1}+\Phi_{2C_{1}}\cdot {\tilde{C}}_{1}+\Phi_{2W_{r}}\cdot {\tilde{W}}_{r}\\ & +\Phi_{2B_{2}}\cdot {\tilde{B}}_{2}+\Phi_{2C_{2}}\cdot {\tilde{C}}_{2}+\Phi_{2W_{ro}}\cdot {\tilde{W}}_{ro}+O_{h} \end{array}$$

where $O_{h}$ represents the higher-order term and the first-order partial derivatives in (29) can be expressed in the following matrix forms:

(30) $$\Phi_{2B_{1}}={\left[\begin{array}{cccc} \frac{\partial \phi_{21}}{\partial B_{1}} & \frac{\partial \phi_{22}}{\partial B_{1}} & \cdots & \frac{\partial \phi_{2l}}{\partial B_{1}} \end{array}\right]}^{T}$$

(31) $$\Phi_{2C_{1}}={\left[\begin{array}{cccc} \frac{\partial \phi_{21}}{\partial C_{1}} & \frac{\partial \phi_{22}}{\partial C_{1}} & \cdots & \frac{\partial \phi_{2l}}{\partial C_{1}} \end{array}\right]}^{T}$$

(32) $$\Phi_{2W_{r}}={\left[\begin{array}{cccc} \frac{\partial \phi_{21}}{\partial W_{r}} & \frac{\partial \phi_{22}}{\partial W_{r}} & \cdots & \frac{\partial \phi_{2l}}{\partial W_{r}} \end{array}\right]}^{T}$$

(33) $$\Phi_{2B_{2}}={\left[\begin{array}{cccc} \frac{\partial \phi_{21}}{\partial B_{2}} & \frac{\partial \phi_{22}}{\partial B_{2}} & \cdots & \frac{\partial \phi_{2l}}{\partial B_{2}} \end{array}\right]}^{T}$$

(34) $$\Phi_{2C_{2}}={\left[\begin{array}{cccc} \frac{\partial \phi_{21}}{\partial C_{2}} & \frac{\partial \phi_{22}}{\partial C_{2}} & \cdots & \frac{\partial \phi_{2l}}{\partial C_{2}} \end{array}\right]}^{T}$$

(35) $$\Phi_{2W_{ro}}={\left[\begin{array}{cccc} \frac{\partial \phi_{21}}{\partial W_{ro}} & \frac{\partial \phi_{22}}{\partial W_{ro}} & \cdots & \frac{\partial \phi_{2l}}{\partial W_{ro}} \end{array}\right]}^{T}$$

Then, substituting the expansion (29) into approximation error (28) yields

(36) $$\begin{array}{ll} f(x)-\hat{f}(x) & ={\tilde{W}}^{T}{\hat{\Phi }}_{2}+{\hat{W}}^{T}\Phi_{2C_{1}}{\tilde{C}}_{1}+{\hat{W}}^{T}\Phi_{2C_{2}}{\tilde{C}}_{2}+{\hat{W}}^{T}\Phi_{2B_{1}}{\tilde{B}}_{1}\\ & +{\hat{W}}^{T}\Phi_{2B_{2}}{\tilde{B}}_{2}+{\hat{W}}^{T}\Phi_{2W_{r}}{\tilde{W}}_{r}+{\hat{W}}^{T}\Phi_{2W_{ro}}{\tilde{W}}_{ro}+\Delta_{0} \end{array}$$

where $\Delta_{0}={\hat{W}}^{T}O_{h}+\varepsilon_{o}$ is a lumped higher-order approximation error, which is bounded as $\left|\Delta_{0}\right|\leq \Delta_{d}$.

As shown in Figure 3, the DHLRNN and adaptive projection algorithm are integrated into the controller. According to the real-time estimation information, a new control law is constructed as

(37) $$u=\frac{1}{\hat{F}}(-k(z_{2}-cz_{1})-\hat{f}(x)-c{\dot{z}}_{1}+{\ddot{v}}_{ref}+\ddot{p}-h(s+\beta \mathrm{sgn}(s))-\eta \mathrm{sgn}(s))$$

where $\eta >\Delta_{d}$, $\hat{F}$ is the estimated value of the control coefficient F.

**Theorem** **2.** *For the dynamic model of the buck converter (3) with external disturbance and parameter variations, if the sliding surface is selected as (24), the control law is designed as (37), the adaptive laws of DHLRNN are designed as (38)–(44) and the adaptive projection algorithm is designed as (45), then the stability of the closed-loop system can be guaranteed, and the voltage tracking error will converge to zero in finite time*.

(38) $$\dot{\tilde{W}}=-\eta_{1}s{\hat{\Phi }}_{2}$$

(39) $${\dot{\tilde{B}}}_{1}^{T}=-\eta_{2}s{\hat{W}}^{T}\Phi_{2B_{1}}$$

(40) $${\dot{\tilde{C}}}_{1}^{T}=-\eta_{3}s{\hat{W}}^{T}\Phi_{2C_{1}}$$

(41) $${\dot{\tilde{W}}}_{r}^{T}=-\eta_{4}s{\hat{W}}^{T}\Phi_{2W_{r}}$$

(42) $${\dot{\tilde{B}}}_{2}^{T}=-\eta_{5}s{\hat{W}}^{T}\Phi_{2B_{2}}$$

(43) $${\dot{\tilde{C}}}_{2}^{T}=-\eta_{6}s{\hat{W}}^{T}\Phi_{2C_{2}}$$

(44) $${\dot{\tilde{W}}}_{ro}^{T}=-\eta_{7}s{\hat{W}}^{T}\Phi_{2W_{ro}}$$

(45) $$\dot{\hat{F}}={\mathrm{Proj}}_{\hat{F}}(\gamma su)$$

**Remark** **4.** *The adaptive projection algorithm [31] in (45) is expressed as*

(46) $${\mathrm{Proj}}_{\hat{F}}(\cdot )=\{\begin{array}{cc} 0 & \hat{F}\geq F_{a}\;\mathrm{and}\;\cdot \;>\;0\\ 0 & \hat{F}\leq F_{i}\;\mathrm{and}\;\cdot \;<\;0\\ \cdot & \mathrm{otherwise} \end{array}$$

*When* $\hat{F}\geq F_{a}$ *and tends to increase, then* $\dot{\hat{F}}=0$, *i.e., the value of* $\hat{F}$ *remains unchanged. On the other hand, when* $\hat{F}\leq F_{i}$ *and tends to decrease, then* $\dot{\hat{F}}=0$, *i.e., the value of* $\hat{F}$ *remains unchanged. Therefore, this algorithm can ensure that* $\hat{F}$ *belongs to a bounded region, thus obtaining the stability of the control signal*.

**Proof** **of** **Theorem** **2.** A new Lyapunov function is defined as follows:

(47) $$\begin{array}{ll} V & =\frac{1}{2}z_{1}^{2}+\frac{1}{2}s^{2}+\frac{1}{2\eta_{1}}{\tilde{W}}^{T}\tilde{W}+\frac{1}{2\eta_{2}}{\tilde{B}}_{1}^{T}{\tilde{B}}_{1}+\frac{1}{2\eta_{3}}{\tilde{C}}_{1}^{T}{\tilde{C}}_{1}+\frac{1}{2\eta_{4}}{\tilde{W}}_{r}^{T}{\tilde{W}}_{r}\\ & +\frac{1}{2\eta_{5}}{\tilde{B}}_{2}^{T}{\tilde{B}}_{2}+\frac{1}{2\eta_{6}}{\tilde{C}}_{2}^{T}{\tilde{C}}_{2}+\frac{1}{2\eta_{7}}{\tilde{W}}_{ro}^{T}{\tilde{W}}_{ro}+\frac{1}{2\gamma }{\tilde{F}}^{2} \end{array}$$

where $\eta_{1},\eta_{2},\eta_{3},\eta_{4},\eta_{5},\eta_{6},\eta_{7},\gamma$ are the parameter learning rates.

Taking the derivative of the Lyapunov function (47) and combining it with (25), it can be obtained that

(48) $$\begin{array}{ll} \dot{V}= & -cz_{1}^{2}+z_{1}z_{2}+s(k(z_{2}-cz_{1})+f(x)+\tilde{F}u+\hat{F}u+c{\dot{z}}_{1}-{\ddot{v}}_{ref}-\ddot{p})\\ & +\frac{1}{\eta_{1}}{\tilde{W}}^{T}\dot{\tilde{W}}+\frac{1}{\eta_{2}}{\dot{\tilde{B}}}_{1}^{T}{\tilde{B}}_{1}+\frac{1}{\eta_{3}}{\dot{\tilde{C}}}_{1}^{T}{\tilde{C}}_{1}+\frac{1}{\eta_{4}}{\dot{\tilde{W}}}_{r}^{T}{\tilde{W}}_{r}+\frac{1}{\eta_{5}}{\dot{\tilde{B}}}_{2}^{T}{\tilde{B}}_{2}+\frac{1}{\eta_{6}}{\dot{\tilde{C}}}_{2}^{T}{\tilde{C}}_{2}\\ & +\frac{1}{\eta_{7}}{\dot{\tilde{W}}}_{ro}^{T}{\tilde{W}}_{ro}-\frac{1}{\gamma }\tilde{F}\dot{\hat{F}} \end{array}$$

where $\tilde{F}=F-\hat{F}$.

Substituting the control signal (37) into (48) yields

(49) $$\begin{array}{ll} \dot{V}= & -cz_{1}^{2}+z_{1}z_{2}+s(f(x)-\hat{f}(x)+\tilde{F}u\\ & -h(s+\beta \mathrm{sgn}(s))-\eta \mathrm{sgn}(s))+H \end{array}$$

Considering the approximation error of DHLRNN in (36), then substituting (36) into (49) obtains

(50) $$\begin{array}{ll} \dot{V}= & -cz_{1}^{2}+z_{1}z_{2}+\tilde{F}su+{\tilde{W}}^{T}{\hat{\Phi }}_{2}s+{\hat{W}}^{T}\Phi_{2B_{1}}{\tilde{B}}_{1}s\\ & +{\hat{W}}^{T}\Phi_{2C_{1}}{\tilde{C}}_{1}s+{\hat{W}}^{T}\Phi_{2W_{r}}{\tilde{W}}_{r}s+{\hat{W}}^{T}\Phi_{2B_{2}}{\tilde{B}}_{2}s\\ & +{\hat{W}}^{T}\Phi_{2C_{2}}{\tilde{C}}_{2}s+{\hat{W}}^{T}\Phi_{2W_{\mathrm{ro}}}{\tilde{W}}_{ro}s-hs^{2}-h\beta \left|s\right|\\ & +\Delta_{o}s-\eta \left|s\right|+H \end{array}$$

By combining with the above adaptive laws of neural network and adaptive projection algorithm, (50) can be further derived as

(51) $$\dot{V}\leq -cz_{1}^{2}+z_{1}z_{2}-hs^{2}-h\beta \left|s\right|+\Delta_{o}s-\eta \left|s\right|$$

According to the analysis in (15)–(17) and the condition of $\eta >\Delta_{d}$, the following inequality holds:

(52) $$\dot{V}\leq -z^{T}Qz-h\beta \left|s\right|\leq 0$$

The condition of |Q|>0 can be ensured through choosing h, c and k. Hence, according to the Lyapunov stability theorem and LaSalle invariance principle, the stability of the closed-loop system can be guaranteed. When t→∞, then $z_{1}\to 0$, s→0. Moreover, Remarks 5–7 are described in the following to demonstrate the finite-time convergence property of the converter system. □

**Remark** **5.** *According to the Remark 2, it can be concluded that* p(0)=e(0), $\dot{p}(0)=\dot{e}(0)$. *Therefore, the following expression can be obtained:*

(53) $$\begin{array}{ll} s(0) & =kz_{1}(0)+z_{2}(0)\\ & =k(e(0)-p(0))+(\dot{e}(0)-\dot{p}(0))+c(e(0)-p(0))\\ & =0 \end{array}$$

*The initial state of the system is already on the sliding mode surface, and it has been proved that* s(t)→0 *as* t→∞, *which means that the reaching condition of sliding mode is eliminated*.

**Remark** **6.** *Considering the sliding surface (24) and Remark 4, let* δ(t)=E(t)−P(t), *yielding*

(54) $$\begin{array}{ll} s & =C(E(t)-P(t))\\ & =C\delta (t) \end{array}$$

*where* $C=\left[\begin{array}{cc} k+c & 1 \end{array}\right]$ *and* k+c>0 *satisfy the Hurwitz condition*. $E(t)={\left[\begin{array}{cc} e(t) & \dot{e}(t) \end{array}\right]}^{T}$ *is the error vector and* $P(t)={\left[\begin{array}{cc} p(t) & \dot{p}(t) \end{array}\right]}^{T}$ *is the terminal function vector*.

*Since the system already possesses global robustness, i.e.,* s=0, *if taking* P(t)=0(∀t≥T), *the tracking error of the system* E(t)(∀t≥T) *will converge to zero in finite time*.

**Remark** **7.** *According to Remark 2, when* t=T, *the condition of* p(T)=0, $\dot{p}(T)=0$, $\ddot{p}(T)=0$ *needs to be satisfied. Hence, the coefficients of the terminal function* p(t) *can be calculated as*

(55) $$p(T)=0\to \left\{ \begin{array}{l} a_{00}=-10\\ a_{10}=15\\ a_{20}=-6 \end{array}\right.$$

(56) $$\dot{p}(T)=0\to \left\{ \begin{array}{l} a_{01}=-6\\ a_{11}=8\\ a_{21}=-3 \end{array}\right.$$

(57) $$\ddot{p}(T)=0\to \left\{ \begin{array}{l} a_{02}=-1.5\\ a_{12}=1.5\\ a_{22}=-0.5 \end{array}\right.$$

**Remark** **8.** *Some limitations still exist in the proposed control method when compared with the conventional technique. Firstly, all parameters of the proposed neural network are updated online through the adaptive laws, which can realize optimal regulation and ensure the stability of a closed-loop system. However, full-parameter learning may cause a high calculation burden. Moreover, in this approach, the detailed network structure is determined by continuous trial and error in advance. If the number of neurons is chosen too large, it will also bring a high calculation burden. Therefore, how to reduce the calculation burden and improve algorithm efficiency by using advanced control strategies will be analyzed in future work*.

## 4. Experimental Results

**Figure 4:**
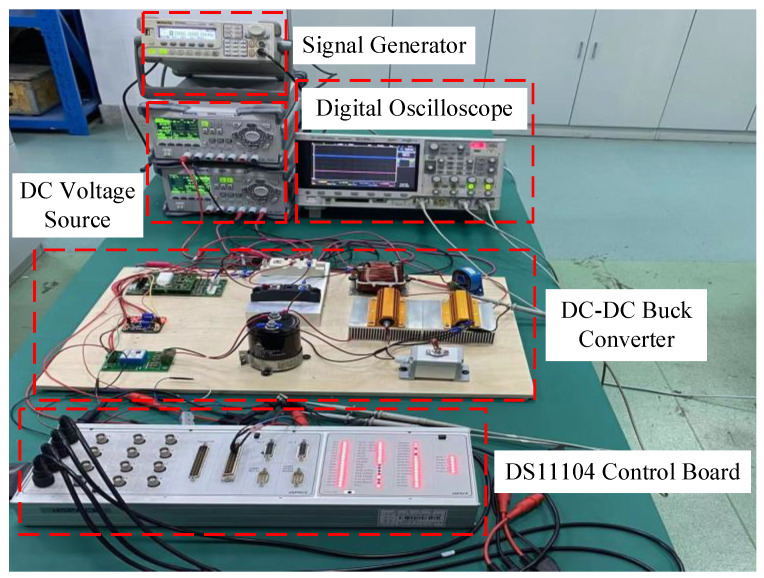
Experimental prototype of the DC-DC buck converter.

**Figure 5:**
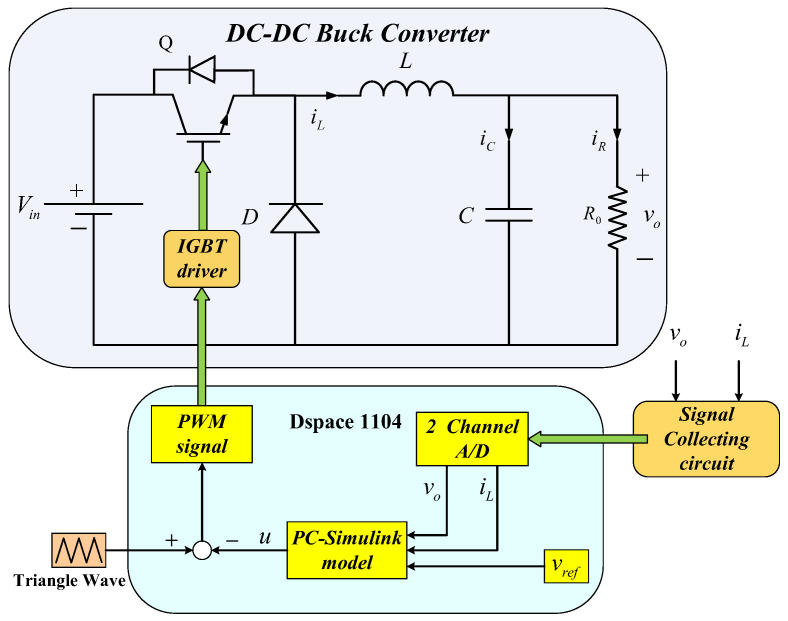
The structure diagram of the experimental platform.

In this section, an experimental prototype is built to illustrate the proposed control method, which is depicted in Figure 4. The experimental prototype comprises a dSPACE-based controller, converter main circuit, signal collecting circuit, DC-supplied voltage and digital oscilloscope. The real-time interface circuit is built by MATLAB/Simulink. The signal-collecting circuit includes the Hall current sensor and the Hall voltage sensor. They acquire voltage signals and current signals, respectively, and the collected signals are sent to the dSPACE through ADC ports. Then, dSPACE calculates the control signal; the obtained control signal is connected to the drive circuit through the PWM output ports to activate IGBT, thus realizing the control of the whole circuit. Moreover, the output waveforms are recorded by the digital oscilloscope DSO-X3034A. The switching frequency for the driving signal is selected as 10 kHz, and the sampling period is selected as $1.5\times 10^{-4}\;s$. The structure diagram of the experimental platform is provided in Figure 5.

The nominal parameter values of the main circuit are listed in Table 1. To demonstrate the effectiveness of the proposed control method, the following two methods are proposed for comparison, including the ideal ABTSMC method and backstepping sliding mode control based on RBFNN (BSMC-RBFNN), where the RBFNN is also utilized to estimate nonlinear function.

To ensure a fair comparison, the above controllers are implemented on identical test conditions. The controller parameters are carefully selected, as listed in Table 2.

**Remark** **9.** *It is worth mentioning that the signals collected by sensors will be reduced due to the turn ratio between the input port and output port. Therefore, the signals sent to the dSPACE are reduced. In order to eliminate this effect, in the real-time interface circuit, the gain modules are introduced, and their value is the same as the turn ratio. Thus, the collected signal can be restored, and the calculation of the control signal will be corrected. Moreover, the ADC and DAC ports often possess non-idealities. These uncertain behaviors will be counteracted by the SMC due to its immunity towards disturbances so that the system can obtain stronger robustness and better disturbance rejection performance*.

**Figure 6:**
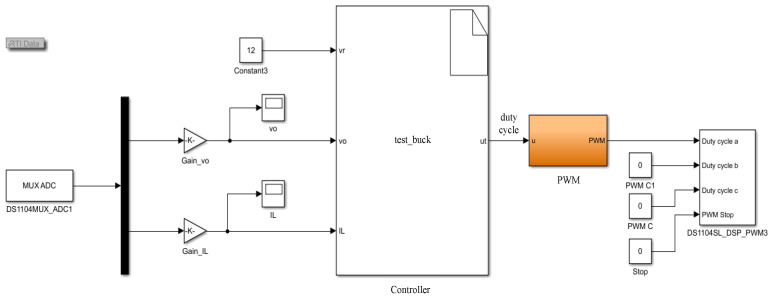
The real-time interface model of the buck converter system.

**Figure 7:**
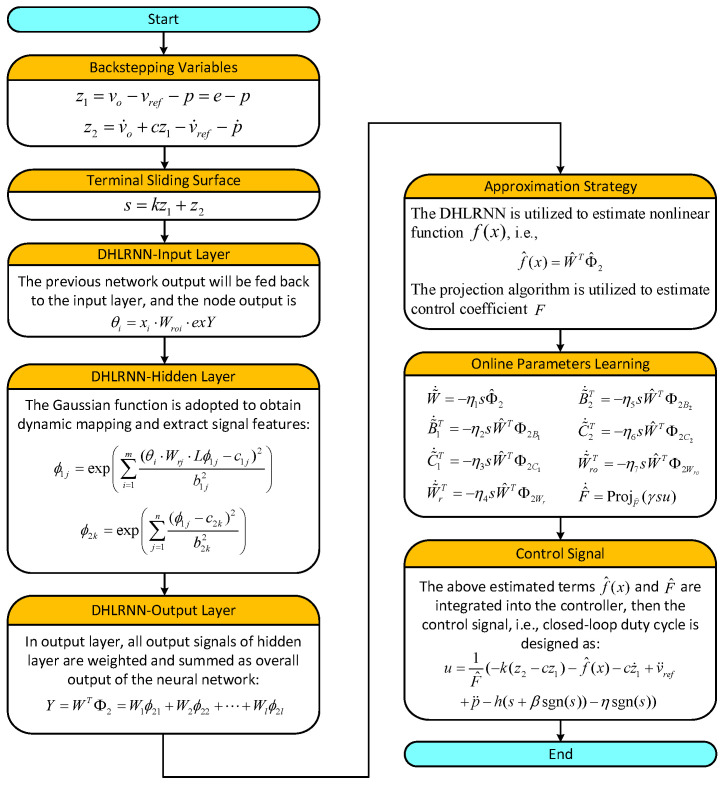
Flowchart of the proposed control method in dSPACE.

**Remark** **10.** *To clearly present the generation process of the closed-loop duty cycle of the converter system, the real-time interface (RTI) model is designed in Figure 6. Firstly, the MUX_ADC module receives the voltage and current signals from the converter circuit. Secondly, according to the reference voltage value, the collected signals and the proposed algorithm, the control signal (37), i.e., duty cycle, can be generated by compiling the main controller module. Then, in the PWM module, the obtained duty cycle is compared with a triangle signal to generate a PWM driving signal. Finally, the driving signal is connected to the IGBT driver through the PWM output module. Moreover, the flowchart of the proposed control method is shown in Figure 7, where the operating steps of the control algorithm in the main controller module are presented*.

**Figure 8:**
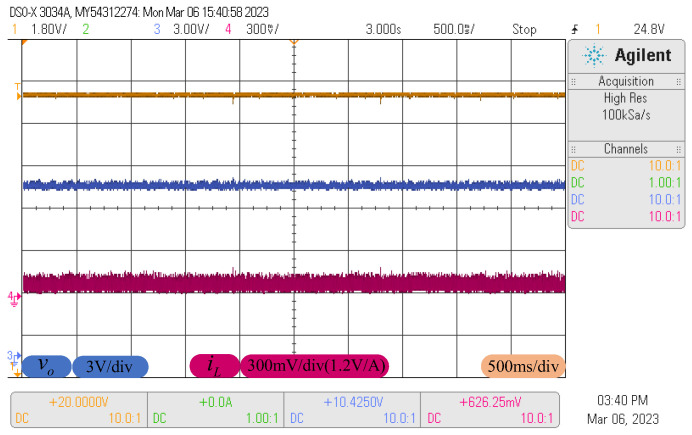
The response curves at the steady-state process.

In normal conditions, the proposed control method is applied to the buck converter. The steady-state response curves are depicted in Figure 8, where the three curves represent input voltage (golden curve), output voltage (blue curve) and inductor current (red curve), respectively. The corresponding waveform descriptions are given at the bottom. It can be seen that output voltage and inductor current are stabilized to the desired value without obvious rise or drop, revealing that the converter system possesses high steady-state accuracy under the proposed control method.

**Figure 9:**
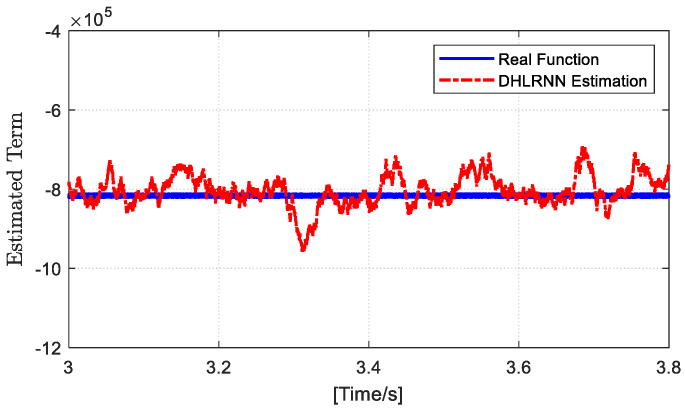
The comparison results of the real function and the DHLRNN estimation.

Since the DHLRNN proposed in this paper is utilized to estimate the nonlinear function, in order to further enrich the experimental results, the comparison results of the real function and the DHLRNN estimation are shown in Figure 9. From Figure 9, it can be concluded that the DHLRNN provides a high estimation precision, which can effectively compensate for the system uncertainty by introducing the estimated value into the controller.

The following conditions are carried out to verify the performance of the above controllers.

### 4.1. Start-up Phase Analysis

**Figure 10:**
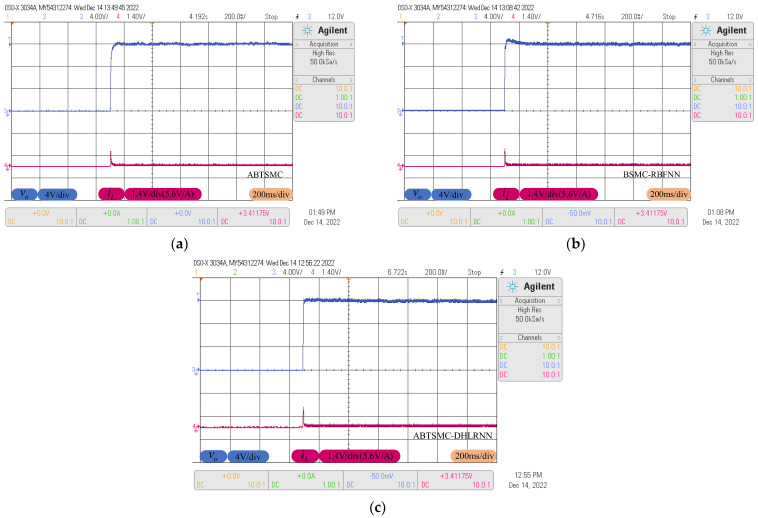
Response curves at the start-up phase. (**a**) ABTSMC. (**b**) BSMC-RBFNN. (**c**) ABTSMC-DHLRNN.

In normal conditions, the response curves obtained for output voltage and inductor current during start-up are shown in Figure 10a–c. As can be seen in Figure 10, the BSMC-RBFNN method produces obvious voltage overshoot during start-up, the peak voltage is about 0.7 V higher than the reference voltage, and the settling time is longer than other control strategies. This is mainly because the lower compensation accuracy of the RBFNN and the BSMC fails to ensure finite-time convergence of the voltage tracking error. Although the ABTSMC method can obtain accurate tracking without obvious voltage overshoot, it takes about 75 ms to stabilize the output voltage through SMC alone. On the contrary, the proposed control method tracks the reference trajectory in 28 ms but with absolutely no overshoot. It is fully confirmed that the TSMC can accelerate the convergence rate of the system, and the DHLRNN can effectively compensate for the system uncertainty.

### 4.2. Load Resistance Variations

**Figure 11:**
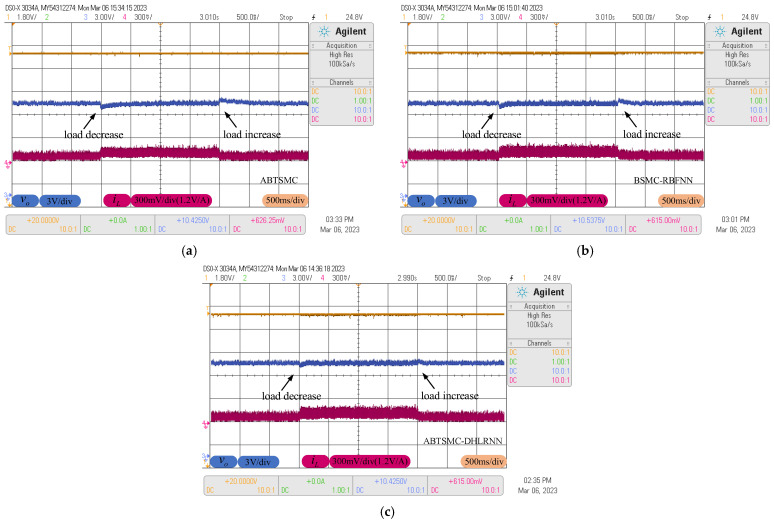
Response curves with load resistance variations. (**a**) ABTSMC. (**b**) BSMC-RBFNN. (**c**) ABTSMC-DHLRNN.

In order to investigate the robustness of the above controllers against the load resistance variations, the buck converter is subjected to a sudden change in load resistance. The load resistance is switched at the value of 30Ω and 20Ω. Figure 11 shows the responses of $v_{o}$ and $i_{L}$ under such a change. As can be seen in Figure 11, the ABTSMC method fails to obtain satisfactory performance when facing load variations, which provides a larger voltage drop/rise and longer settling time. Although BSMC-RBFNN method can provide a faster response when the estimated term is introduced into the controller, it needs about 210 ms to eliminate the effect of load resistance variations due to its slow learning, and the voltage drop/rise is about 0.5 V, whereas the proposed control method presents slight rise/drop in output voltage ripple around the reference value, and the settling time is much shorter than other control algorithms, revealing stronger robustness and better disturbance rejection performance by the proposed control method in rejecting load resistance variations.

### 4.3. Reference Voltage Variations

**Figure 12:**
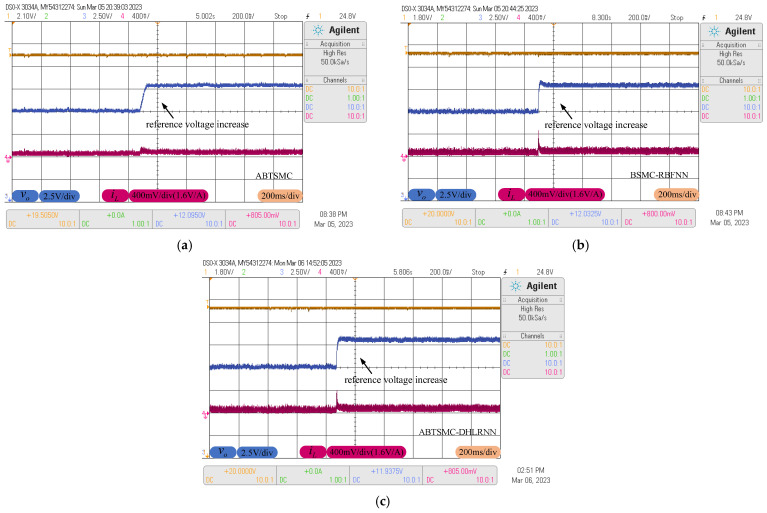
Response curves with reference voltage variations. (**a**) ABTSMC. (**b**) BSMC-RBFNN. (**c**) ABTSMC-DHLRNN.

To examine the tracking performance of each controller during reference variations, Figure 12 shows the tracking behaviors of the output voltage and inductor current, where the reference voltage occurs a sudden change from the nominal value of 12 V to 15 V. From Figure 12, during the occurrence of such uncertainty, the ABTSMC method takes about 90 m to track the new trajectory of 15 V due to the lack of the estimation of the uncertain terms, while the BSMC-RBFNN method is found to track the new reference voltage in 78 ms, but it produces about 0.6 V voltage overshoot. The proposed control method is comparatively faster to reject such uncertainty, which can smoothly track the new reference trajectory in 40 ms. Due to the high compensation accuracy of the DHLRNN, this method provides better voltage tracking performance with reference voltage variations.

### 4.4. Input Voltage Fluctuations

**Figure 13:**
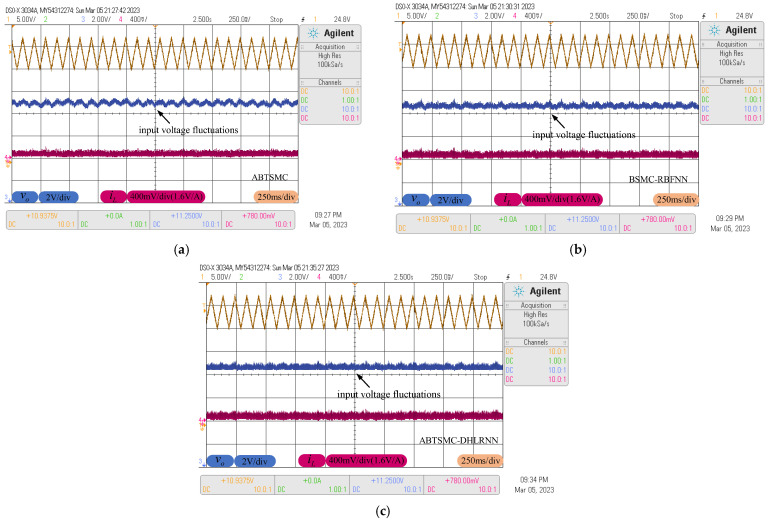
Response curves with input voltage fluctuations. (**a**) ABTSMC. (**b**) BSMC-RBFNN. (**c**) ABTSMC-DHLRNN.

A triangle disturbance (period 100 ms, amplitude 2 V) is introduced to approximate the input voltage fluctuations. The dynamic processes of the above controllers in the presence of time-varying disturbance are plotted in Figure 13, where the golden curve is the real input voltage. It can be seen that the ABTSMC method fails to ensure smooth tracking curves; the corresponding voltage fluctuations are clearly visible, which will significantly affect the stability of the output voltage. The BSMC-RBFNN method can provide a smaller fluctuation amplitude, but it cannot completely eliminate the adverse effect of the time-varying disturbance. However, since the DHLRNN and adaptive projection algorithm can continuously adjust output values according to the system dynamics, the ABTSMC-DHLRNN method provides higher tracking accuracy with the smallest voltage fluctuations, and the relevant output voltage waveform almost presents a straight line.

### 4.5. Comparison Analysis and Summary

Table 3 shows the performance indices of maximum voltage rise/maximum voltage drop (MVR/MVD) and settling time (ST) of the above controllers. The proposed control method adopts two estimation techniques to counteract and compensate for the system uncertainty caused by parameter variations, and the DHLRNN possesses higher estimation and compensation accuracy than the RBFNN with a single hidden layer due to the design of multiple hidden layers and neuron feedback loop. Thus, the stability and disturbance rejection performance of the converter system can be improved during the tracking process. Moreover, the TSMC can further accelerate the system convergence in comparison with the conventional SMC, which ensures a shorter settling time. The corresponding comparison results in Table 3 illustrate that the proposed control method possesses higher tracking accuracy and faster dynamic response.

To sum up, the proposed control method comprises TSMC, which can ensure the finite-time convergence of the tracking error. Thus, the converter system obtains a faster transient response during start-up, which takes a shorter start-up time than other control algorithms. Secondly, since the proposed control method adopts two estimation techniques to approximate nonlinear function and control coefficient, the model uncertainty caused by parameter variations can be effectively compensated and counteracted by combining with precious estimation information. Thus, the proposed converter obtains better disturbance rejection ability in the presence of load variations and reference voltage variations, and the corresponding output waveforms provide shorter tracking time with absolutely no overshoot. Moreover, due to the approximation capability of the DHLRNN and the discontinuous property of the switching term when faced with the time-varying disturbance caused by input voltage fluctuations, the proposed controller presents higher tracking accuracy, and the corresponding output waveform provides smoother curves.

## 5. Conclusions

In order to improve the stability of the DC power, a composite ABTSMC method with DHLRNN is proposed to realize the voltage tracking control of the DC-DC buck converter. This method adopts the DHLRNN and adaptive projection algorithm to estimate nonlinear function and control coefficient, and the adverse effect of system uncertainty can be effectively eliminated by introducing the above estimates into the controller. Moreover, the switching term of the sliding mode can also be reduced, and thus, the chattering phenomenon is alleviated. To sum up, due to the design of the double hidden layer structure and neuron feedback loop, the proposed neural network can obtain higher estimation accuracy with fewer neurons. The neural network parameters are trained online by using the adaptive laws to obtain optimal values. The adaptive projection algorithm can ensure that the estimated term belongs to a bounded region, and the stability of the control signal can be obtained. Since the backstepping terminal sliding mode control combines the merits of the backstepping design and TSMC, it can not only reduce the complexity of the control design but also obtain the finite-time convergence property of the tracking error. Experimental results demonstrate the superiority of the proposed control method under multiple operating conditions. Therefore, this method has a good application prospect in the DC-DC buck converter.

## Figures and Tables

**Table 1:** Nominal circuit parameter values.

Description	Parameter	Value	Units
Inductor	L	6	mH
Capacitor	C	2200	uF
Load resistance	$R_{0}$	30	Ω
Input voltage	$V_{in}$	25	V
Reference voltage	$v_{ref}$	12	V

**Table 2:** Controller parameter values.

Controllers	Parameters and Values
ABTSMC	$c=2\times 10^{5}$ ,k=4000 ,h=2000 ,β=1200 ,η=0.1 ,T=0.01
BSMC-RBFNN	$c=2\times 10^{5}$ ,k=4000 ,h=100 ,β=120 ,η=0.1
ABTSMC-DHLRNN	$c=2\times 10^{5}$ ,k=4000 ,h=2000 ,β=1200 ,η=0.1 ,T=0.01

**Table 3:** Performance indices comparison in the above tests.

Test	Controllers	Performance Indices	
		MVR/MVD (V)	ST (ms)
1	ABTSMC	−/−	75/−
	BSMC-RBFNN	0.7/−	160/−
	ABTSMC-DHLRNN	−/−	28/−
2	ABTSMC	0.6/0.6	400/400
	BSMC-RBFNN	0.5/0.5	210/206
	ABTSMC-DHLRNN	0.3/0.35	125/170
3	ABTSMC	−/−	90/−
	BSMC-RBFNN	0.6/−	78/−
	ABTSMC-DHLRNN	−/−	40/−
4	ABTSMC	0.4/0.4	−/−
	BSMC-RBFNN	0.3/0.4	−/−
	ABTSMC-DHLRNN	0.3/0.2	−/−

## Data Availability

Not applicable.
